# A C-band broadband ortho-mode transducer for radioastronomy polarimetry

**DOI:** 10.1186/s40064-016-3761-5

**Published:** 2016-12-02

**Authors:** Ivan S. Ferreira, Camilo Tello, Miguel Bergano, Thyrso Villela, Domingos Barbosa, George F. Smoot

**Affiliations:** 1 Laboratório de Cosmologia e Astrofísica, Instituto de Física, Universidade de Brasília, Campus Universitário Darcy Ribeiro, Asa Norte, Brasília, 70919-970 Brazil; 2 Divisão de Astrofísica – DAS, Instituto Nacional de Pesquisas Espaciais (INPE), Av. dos Astronautas, 1.758, Jd. Granja, São José dos Campos, SP CEP 12227-010 Brazil; 3 Wiseware Engineered Solutions, Zona Industrial da Mota, Rua 12, Lote 51, Fração E, 3830-527 Gafanha da Encarnação, Portugal; 4 ENGAGE SKA, Instituto de Telecomunicações, Campus Universitário de Santiago, 3810-193 Aveiro, Portugal; 5 Lawrence Berkeley National Laboratory, 1 Cyclotron Road, MS 50-5005, Berkeley, CA 94720 USA; 6 Université Paris-Diderot APC, Bâtiment Condorcet, 10 rue Alice Domon et Léonie Duquet, 75205 Paris Cedex 13, France

**Keywords:** Radioastronomy, Polarimeters, Ortho-mode transducers

## Abstract

**Background:**

We describe the design, the construction and performance of a narrow band ortho-mode transducer, currently used in the 5 GHz polarimetric receiver of the Galactic Emission Mapping project.

**Results:**

The ortho-mode transducer was designed to achieve a high degree of transmission within the 400 MHz of the GEM band around the 5 GHz (4.8–5.2 GHz). It is composed of a circular-to-square waveguide transition, a septum polarizer, a thin waveguide coupler and a smooth square-to-rectangular waveguide transition with custom waveguide bends to the output ports.

**Conclusion:**

Our simulations and measurements show a very low level of cross-polarization of about −60 dB and a good impedance match for all three ports (S11; S22; S33 < −30 dB) with only 0:25 dB of insertion loss offset across the 400 MHz (4.8–5.2 GHz) of the reception bandwidth.

## Background

The key component of most polarimeters is the ortho-mode transducer (OMT), which splits the orthogonal polarization modes of the incoming sky radiation into two or more balanced RF chains. There are many ways to build an OMT [see Bøifot (1991) for a review], but all of them require a careful analysis of the symmetries of the propagation modes inside the square, rectangular and circular waveguide sections of any particular type of OMT design. The main requirement for an OMT used in radioastronomy experiment dedicated to reveal polarized patterns of the Galactic radio continuum, like the Galactic Emission Mapping (GEM) project (Torres et al. 1996), is a high isolation between the output ports across the intended bandwidth. A cross-talk between the output ports of the OMT can generate several undesired features in the radio sky map, invalidating the survey. Other properties of the OMT, like return loss and insertion loss offsets, will also affect the sky map, in particular by reducing its pixel sensitivity.

The main goal of the GEM project is to characterize the Galactic emission in total intensity and polarization between 408 MHz and 10 GHz, by producing astrophysical foreground templates to decontaminate Cosmic Microwave Background Radiation maps (see Tello et al. (2013) for recent results at 2300 MHz). Galactic emission in the GEM frequency bands at 5 and 10 GHz is dominated by synchrotron radiation. In these bands, the radio emission has a high degree of linear polarization and interstellar Faraday effects are still negligible. Thus, these characteristics make those bands a choice of prime importance to improve the foregrounds impact on surveys of cosmological significance like those produced by Planck satellite mission (Bouchet and Gispert 1999; Ade et al. 2014; Adam et al. 2016) and those planned for the next generation CMB space missions like the ESA CoRE proposal (de Zotti et al. 2016). The GEM data at 5 and 10 GHz can also provide useful constraints together with the absolutely calibrated Galactic data from the ARCADE balloon experiment (Fixsen et al. 2011). In this article we describe an OMT that can be classified as Septum-Branching OMT class 1, following the classification presented in (Bøifot 1991; Uher et al. 1993). It was developed for a pseudo-correlation polarimeter, suitable for a bandwidth of 400 MHz centered at 5 GHz and having a measured sensitivity of about 1:6 mk/√s (Bergano et al. 2007).

The OMT should have its best performance in the GEM band (4.8–5.2 GHz). It is coupled to a corrugated feed horn. This system has been used in the focal plane of a Cassegrain 5.5 m dish antenna, which continuously rotate to map the polarized Southern sky from an observational site in Brazil. The observations started in 2006. GEM is followed by a sister counterpart (GEM-P) aiming the coverage of the Northern sky (Barbosa et al. 2006; Tello et al. 2013). Other important surveys include the C-Band All Sky Survey (CBASS) (Irfan et al. 2015; King et al. 2010) that is currently acquiring data, having achieved a first Northern Sky intensity survey at 5 GHz (Irfan et al. 2015). The OMT for the CBASS instrument is described in Grimes et al. (2007) and achieves a performance similar to the GEM’s OMT described here.

## Design and methods

**Fig. 1:**
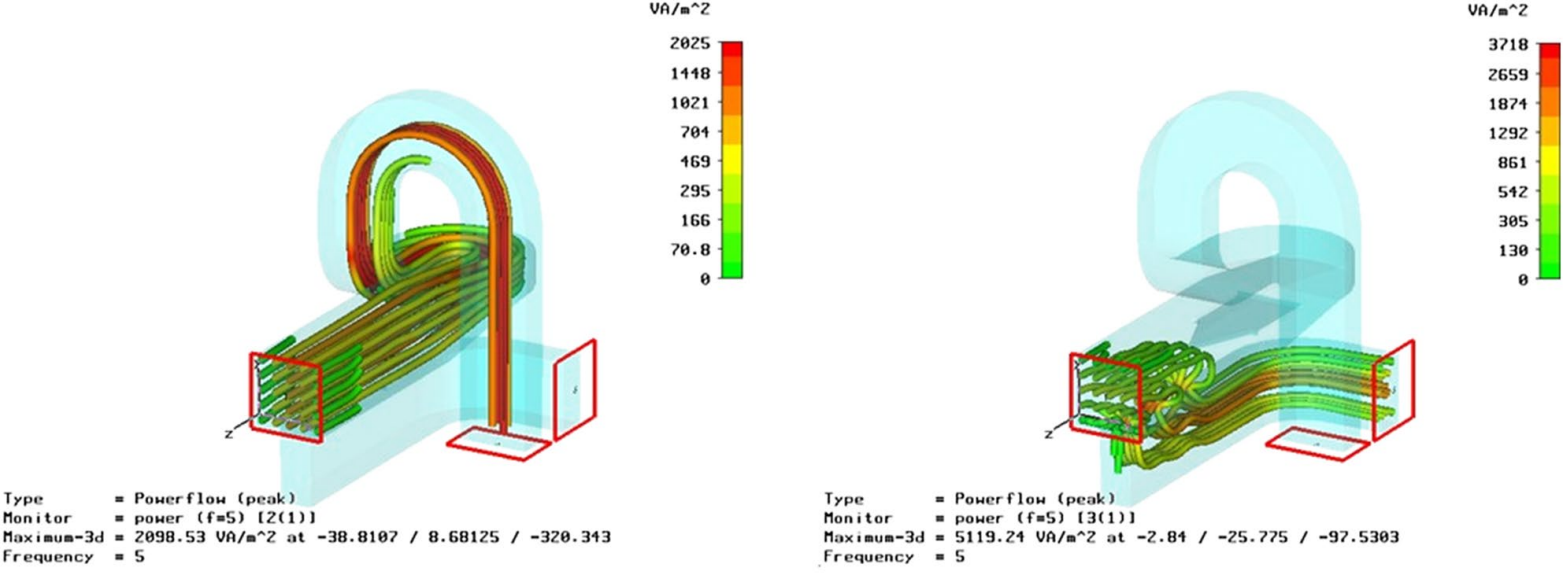
Example of Finite-element simulations of mode propagation for two polarizations

Initially, a classical RF design approach, developed at UC Berkely and similar to CBASS (Grimes et al. 2007) was followed. However, due to its poor polarization purity performance, most likely due to a manufacturing problem, and the lack of space in the 5-m antenna hub (already filled with ancillary systems of the receiver) it was necessary to design a new OMT, more compact and easier to mount between the horn and the receiver. This in turn required additional performance fine tuning and several finite-element simulations (see Fig. 1).

**Fig. 2:**
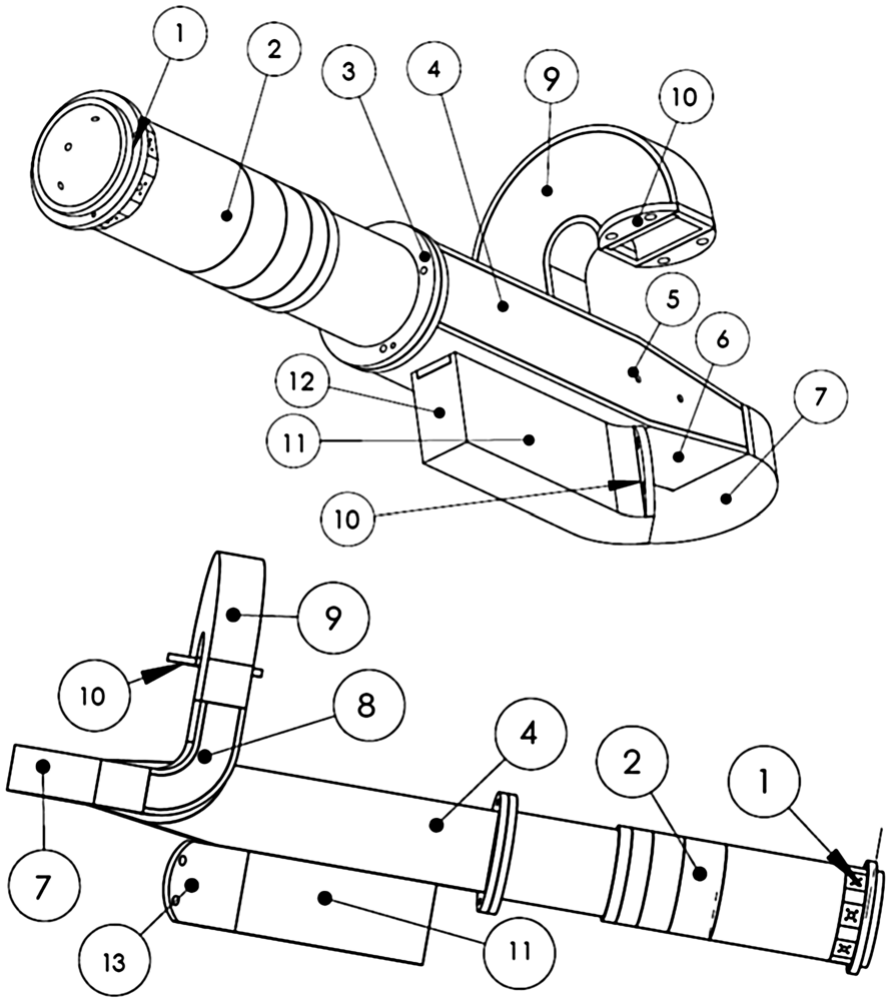
The 5 GHz GEM OMT: *1* the Multiple Injector Noise Source Assembly (MINSA) with circular WG13 waveguide; *2* three-step circular-to square transition; *3* flange between square waveguides; *4* square waveguide with aperture directional coupler; *5* septum support pins; *6* smooth square to-rectangular transition; *7* first 180 ± H-plane bend; *8* 90 ± E-plane bend; *9* second 180 ± H-plane bend; *10* waveguide-to-coax adapter flanges; *11* parallel coupled port; *12* tuning back-short and *13* 45 ± E-plane bend

Hence, a new design consisting of four sections was considered: a circular to-square waveguide transition; an aperture coupler with a septum; a smooth transition between square and rectangular waveguides; and a series of waveguide bends in both ports. The complete OMT assembly is shown in the wire frame model of the waveguide structure in Fig. 2, where the number labels indicate the components of each section.

Each of the four sections is described below: the transition between the circular and the square waveguides was made using three λ_g_ = 4 steps (where λ_g_ stands for the guided wavelength), whose actual length was optimized using numerical simulations based on finite-element analysis (CST Microwave Studio—Computer Simulation Technology GmbH). The same code, henceforth the CST code, was used in the optimization of all parts of the OMT. Figure 1 shows two of the finite-element propagation simulations to check polarization mode propagation and isolation.

**Fig. 3:**
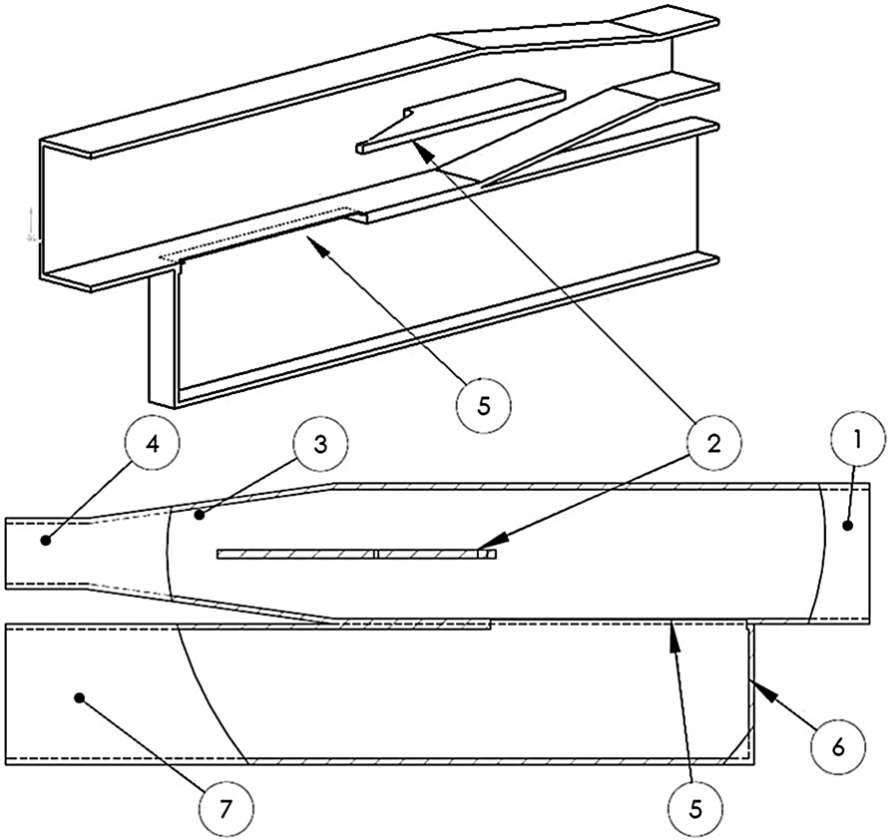
A cut-section view of the main part of the OMT body. Showing: *1* the square waveguide; *2* the septum; *3* smooth square-to-rectangular transition; *4* rectangular waveguide; *5* the aperture directional coupler and *6* tuning back-short

The polarization splitter, the main component of the OMT, is shown in Fig. 3. It achieves its basic functionality via a directional coupler and a septum. The directional coupler is shaped into a finite length coupling slot. A similar device, also called a T-Junction, is typically used to couple waveguides of similar cross-sections (Ludovico 1999), whereas in our OMT we couple a square waveguide from one side to a rectangular WR187 waveguide on the other side. The CST code was used to find the optimal length, width and height of the slot. In fact, the optimal choice for the geometry of this slot was ideal to make a groove in the inside wall of the square waveguide (effectively reduced its thickness to 0.5 mm), where a rectangular aperture (90.0 mm × 5.68 mm) was cleared. As shown in the literature (Uher et al. 1993), the phase difference introduced by this kind of coupling is 90°.

A sliding back-short closes the WR187 waveguide to enable the transmission tuning of the OMT during bench tests. The septum has its length proportional to the desired rejection level of higher order modes (Uher et al. 1993), but it is limited by the maximum allowed physical size of the OMT inside the dish hub. The small available space of the dish hub, in the back of the antenna was a strong limiting factor, conditioning the adoption of design options similar to the CBASS’ OMT shape (long, cylinder like).

On the other hand, the septum width should be as small as possible to minimize the return loss of the mode that will propagate through it. Similarly, the shape of the septum was carefully chosen, starting with a rectangular septum, passing through a rift wedge-like design, and arriving finally at the arrow-like shape, shown in Fig. 3, to achieve the minimum return loss. In addition to the shape and dimensions of the septum, computer simulations were needed to determine the position of the septum with respect to the slot.

**Fig. 4:**
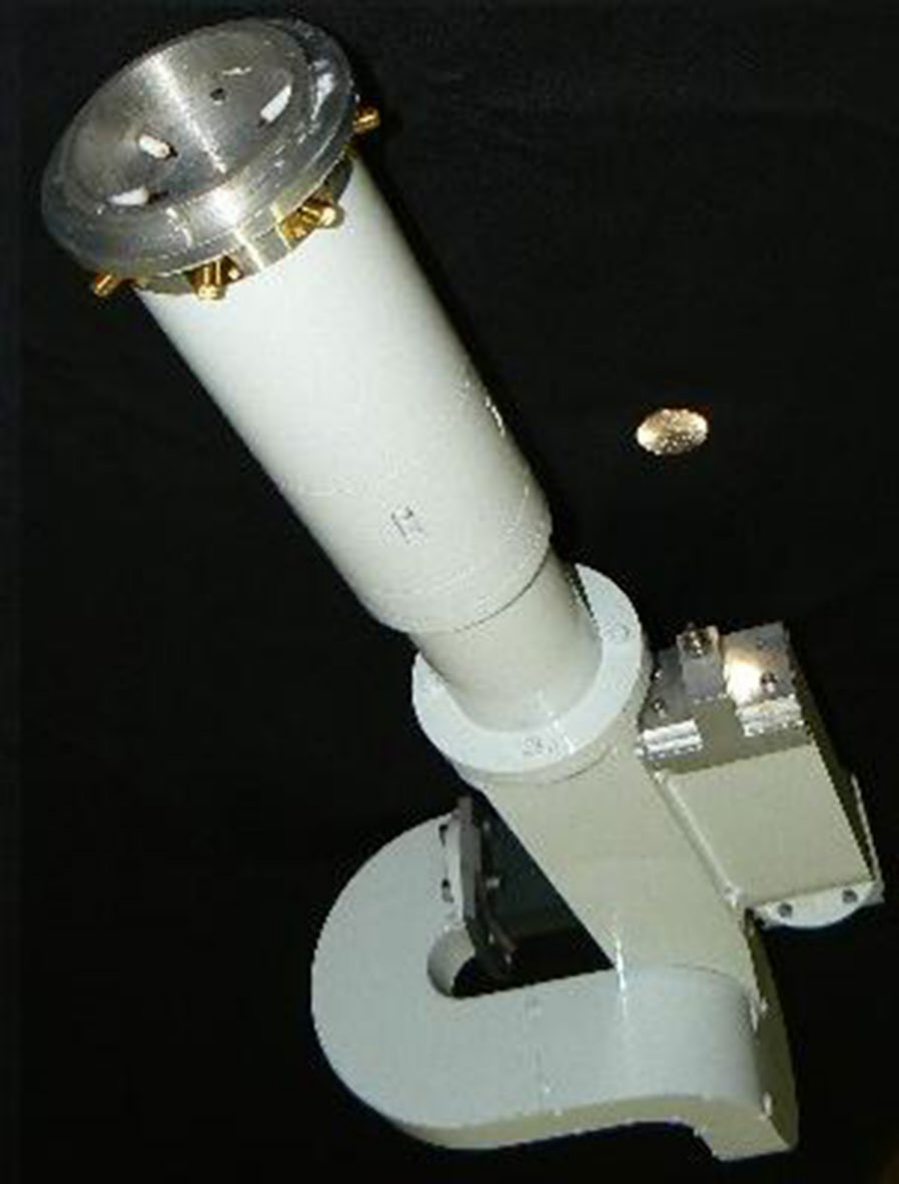
The newly manufactured GEM 5 GHz OMT. A coin is shown for reference

Our optimal solution was that the tip of the septum had to be coincident with the end of the slot. The final width of the septum was 3 mm, and its length was 102.5 mm. After the septum, there is a smooth transition to transform the square waveguide into a rectangular WR187 waveguide. The length of this transition had to be small enough not to exceed the physical size of the OMT, but large enough to reach a maximum acceptable return loss of −40 dB. We achieved this condition by optimizing its length at 86 mm with the septum halfway through into the transition. Due to the physical space where the OMT was installed and the necessity to avoid higher modes of propagation and cross-talk between the ports, we had to extend the WR187 waveguides along several bends. For the waveguide of the coupled parallel port (number 11 in Fig. 2), we introduced a 45° E-plane bend; while for the waveguide extending from the smooth square-to-rectangular transition (number 6 in Fig. 2), we introduced two 180° H-plane bends at both ends of a 90° E-plane bend. All these bends were designed with the aid of the CST code subject to a maximum tolerable return loss of −40 dB. The inner radius of the typical bend was found to be 23.15 mm for an E-plane bend and 18 mm for an H-plane bend. All sections of the OMT were machined from 6061 aluminium alloy; Fig. 4 shows its final construction.

**Fig. 5:**
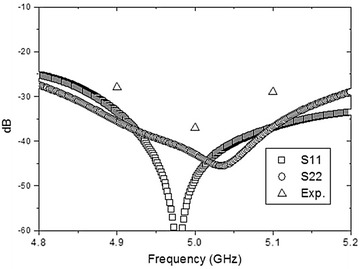
Results for S-parameters from numerical simulations, for both polarization modes (shown as *S11* and *S22*), and from bench measurements (shown as *Exp*), using in both cases the square waveguide as input port

**Fig. 6:**
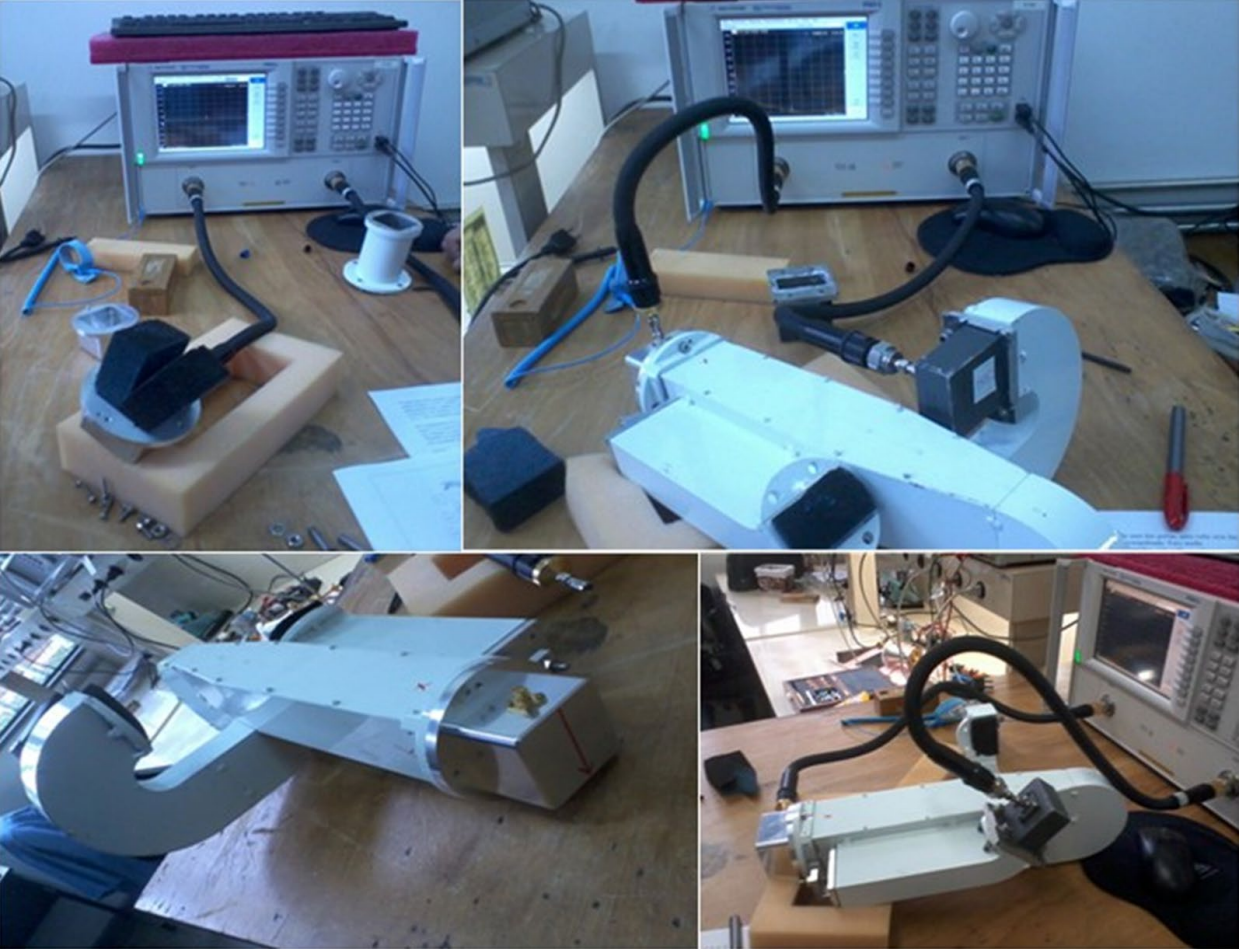
The measurement setup at the laboratory, showing the network analyzer and the port microwave absorbers

The installation of the GEM polarimeter requires the OMT to match the throat section of a corrugated feed horn, which is supported at the vertex of the primary reflector by a latching mechanism. During routine observations, the OMT operates at room temperatures (~300 K), while feeding the cryogenic frontend of the polarimeter housed inside the antenna hub. Finally, Fig. 5 summarizes the results of the computational analysis of the OMT in terms of the S-parameters (S11, S22, S33) along with some experimental points, obtained during bench tests, which we will describe in the following section. Figure 6 depicts the workbench experimental measurement setup.

## Results

The bench tests were conducted at the INPEs’ Aerospace Engineering Division (DEA) laboratory with an Agilent E8362B network analyzer, adjusted with a calibration offset to remove the effect of the cables and their connectors. The measurements of S parameters (basically insertion loss and return loss of the ports) were conducted in two steps. First, we measured the impedance match of the 3-step circular-to-square waveguide transition using a circular waveguide-to coax adapter with a SMA probe in the place of the Multiple Injector Noise Source Assembly (MINSA) assembly shown in Fig. 2. A return loss of about −40 dB was found within 200 MHz of the proposed center frequency of 5 GHz. This return loss was accounted for as an additional calibration offset during the measurement of the overall return loss of the OMT. Second, using the above measurement as a calibration offset, we obtained a return loss below −30 dB for the entire OMT. The measurements for port 1 at 4.9, 5.0, and 5.1 GHz are plotted in Fig. 5 together with simulated profiles for the two possible polarization modes (labelled as S11 and S22). Similar return losses were obtained at the SMA probes of the rectangular waveguide-to-coax adapters labelled 10 in Fig. 2. During the tests, all open ports were filled with microwave absorbers. The experimental points became indistinguishable from the simulation curves for these two polarization modes once the tuning back-short was properly adjusted through several iterative steps.

**Fig. 7:**
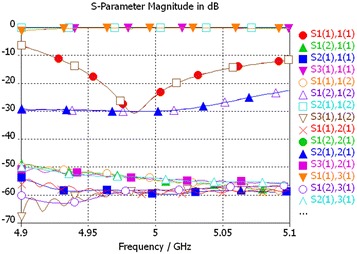
Measurements and simulated curves after the iterative adjustments of the tuning back short

In order to estimate the cross-talk between the outputs of the OMT, we coupled the network analyzer to the SMA probes of the waveguide-to-coax adapters at the output ports of the rectangular waveguides (10 in Fig. 2). The tests showed the level of cross-talk to be below −60 dB. The same upper limit to the level of cross-polarization is reached if the polarized signal is transmitted from port 1 to the output port. The measurements also infer a total phase difference of 120° and an insertion loss offset in 0.25 dB between the output ports. These numbers reflect the use of long and curved rectangular waveguides in order to adjust the dimensions of the OMT to those of the receiver in the enclosing hub. In Fig. 6, we can check the measurement setup and Fig. 7 we can compare the measurements points against the simulated curves, after the iterative adjustments of the tuning back short.

## Conclusion

A high isolation 5 GHz OMT was presented. Its design was the result of a process of optimization that involved no less than 96 different CAD models. The measured insertion loss offset for this OMT was about 0.25 dB between 4.8 and 5.2 GHz, with a cross-polarization level of about −60 dB. There is a total phase difference of 120° between the output signals, to be accounted for by phase shifters included in the RF chain of the polarimeter. These numbers, together with a return loss of −30 dB for all ports, satisfy all the requirements for polarization measurements with the GEM experiment, as well as for other experiments dedicated to survey the microwave polarized sky in the C-band.

## Abbreviations

OMT: orthomode transducer
GEM: Galactic Emission Mapping
MINSA: Multiple Injector Noise Source Assembly
